# Anesthesia for fetal operative procedures: A systematic review

**DOI:** 10.3389/fpain.2022.935427

**Published:** 2022-09-12

**Authors:** Miriam Duci, Rebecca Pulvirenti, Francesco Fascetti Leon, Irma Capolupo, Paola Veronese, Piergiorgio Gamba, Costanza Tognon

**Affiliations:** ^1^ Pediatric Surgery Unit, Department of Women’s and Children’s Health, Padua University Hospital, Padua, Italy; ^2^ Department of Medical and Surgical Neonatology, Bambino Gesù Children’s Hospital, IRCCS, Rome, Italy; ^3^ Maternal-fetal Medicine Unit, Department of Women’s and Children’s Health, Padua University Hospital, Padua, Italy; ^4^ Anesthesiology Pediatric Unit, Department of Women’s and Children’s Health, Padua University Hospital, Padua, Italy

**Keywords:** fetal surgery, perinatal outcome, fetal analgesia, maternal-fetal anesthesia, fetal pain

## Abstract

**Objective:**

The anesthetic management of fetal operative procedures (FOP) is a highly debated topic. Literature on fetal pain perception and response to external stimuli is rapidly expanding. Nonetheless, there is no consensus on the fetal consciousness nor on the instruments to measure pain levels. As a result, no guidelines or clinical recommendations on anesthesia modality during FOP are available. This systematic literature review aimed to collect the available knowledge on the most common fetal interventions, and summarize the reported outcomes for each anesthetic approach. Additional aim was to provide an overall evaluation of the most commonly used anesthetic agents.

**Methods:**

Two systematic literature searches were performed in Embase, Medline, Web of Science Core Collection and Cochrane Central Register of Controlled Trials up to December 2021. To best cover the available evidence, one literature search was mostly focused on fetal surgical procedures; while anesthesia during FOP was the main target for the second search. The following fetal procedures were included: fetal transfusion, laser ablation of placental anastomosis, twin-reversed arterial perfusion treatment, fetoscopic endoluminal tracheal occlusion, thoraco-amniotic shunt, vesico-amniotic shunt, myelomeningocele repair, resection of sacrococcygeal teratoma, ligation of amniotic bands, balloon valvuloplasty/septoplasty, ex-utero intrapartum treatment, and ovarian cyst resection/aspiration. Yielded articles were screened against the same inclusion criteria. Studies reporting anesthesia details and procedures’ outcomes were considered. Descriptive statistical analysis was performed and findings were reported in a narrative manner.

**Results:**

The literature searches yielded 1,679 articles, with 429 being selected for full-text evaluation. A total of 168 articles were included. Overall, no significant differences were found among procedures performed under maternal anesthesia or maternal-fetal anesthesia. Procedures requiring invasive fetal manipulation resulted to be more effective when performed under maternal anesthesia only. Based on the available data, a wide range of anesthetic agents are currently deployed and no consistency has been found neither between centers nor procedures.

**Conclusions:**

This systematic review shows great variance in the anesthetic management during FOP. Further studies, systematically reporting intraoperative fetal monitoring and fetal hormonal responses to external stimuli, are necessary to identify the best anesthetic approach. Additional investigations on pain pathways and fetal pain perception are advisable.

## 1. Introduction

The recent advancement of minimally invasive techniques, together with a deeper knowledge of maternal-fetal physiology, led to major progresses in the field of fetal surgery. As a result, the treatment of congenital malformations, historically planned after delivery, has become feasible at the prenatal stage. Nonetheless, the maternal and fetal anesthetic management during such procedures is still controversial. Indeed, based on the invasiveness of the procedure, either general or regional maternal anesthesia can be required, in conjunction or not with fetal direct anesthesia (1). Generally, fetal operative procedures (FOP) are challenging, and anesthetic care needs to take into account not only maternal and fetal physiology, but also the anesthetic drugs’ interaction with the maternal-fetal health (2). Furthermore, it is still debated whether the fetus is able to experience pain during a fetal procedure. Recent studies describing an increase in cortisol and adrenaline levels or the development of bradycardia after painful stimuli in fetuses of 16–25 weeks of gestational age (GA), demonstrated a reaction to pain during prenatal life (3, 4). Additionally, available evidence on fetal physiology showed that between 16–24 weeks of GA the thalamus, an essential organ for pain perception, seems to be adequately developed (5–7). However, how much these changes imply a conscious pain processing and how to best measure the pain level in fetuses is still poorly understood. No anesthesia guidelines or standardized protocols for FOP are available, and the decision mainly depends on the expert’s opinion and expertise.

The lack of evidence prompted us to perform a systematic review on the use of maternal-fetal anesthesia in FOP. Therefore, this study aimed to collect the available knowledge on the most common fetal interventions and summarize the reported outcomes for each anesthetic approach. For every prenatal intervention, outcomes were compared between procedures performed under maternal anesthesia and those performed under maternal and fetal anesthesia; when applicable to the retrieved data, further distinction among loco-regional and general maternal anesthesia was made. Additional objective was to provide a general evaluation of the most commonly used anesthetics for all the included procedures.

## 2. Methods

This review was performed according to an *a priori* designed protocol and recommended for systematic reviews (8, 9). Additionally, the principles of the “preferred reporting items for systematic reviews” (PRISMA) statement were adhered to (10). This study is registered in the PROSPERO database (registration number CRD42022302979). A systematic literature search was performed in Embase, Medline, Web of Science Core Collection and Cochrane Central Register of Controlled Trials until December 14 2021. The fetal operative procedures considered were: fetal transfusion, laser ablation of placental anastomosis, twin-reversed arterial perfusion treatment, fetoscopic endoluminal tracheal occlusion (FETO) in congenital diaphragmatic hernia (CDH), thoraco-amniotic shunt, vesico-amniotic shunt, myelomeningocele (MMC) repair, resection of sacrococcygeal teratoma, ligation of amniotic bands, balloon valvuloplasty/septoplasty, ex-utero intrapartum treatment (EXIT), and ovarian cyst resection/aspiration. Since combining each procedure with the search term “anesthesia” yielded a limited number of studies, a second search focused on the provision of maternal-fetal anesthesia was performed. Databases screened and search date were the same for both systematic searches. The search strategies are attached in the Supplementary Material. The search and selection criteria were restricted to English language articles and limited to humans. No publication year restriction was considered. Due to the known clinical heterogeneity of included studies a meta-analysis method would have been inappropriate. Therefore, we described our findings in a narrative manner.

### 2.1. Inclusion criteria and exclusion criteria

Studies were assessed according to the following criteria: population characteristics, intervention and reported outcome. All studies describing outcomes for fetal procedures and providing details on maternal-fetal anesthesia were included. Studies describing maternal-fetal anesthesia, but no procedure outcome were only included in the overall analysis of anesthesia modality and in the report of anesthetic drugs used. Since many studies refer to anesthesia and analgesia interchangeably, both terms were considered during the studies’ screening against inclusion criteria.

Conference abstracts, editorials, letters, short surveys, studies reporting non-original data (systematic reviews, meta-analysis, narrative reviews) and unavailable full-text articles were excluded. Absence of discrete patients data was an additional exclusion criteria.

### 2.2. Study selection

Two review authors (MD and RP) independently screened titles and abstracts to select eligible studies. Disagreements about study selection were resolved by discussion. MD and RP screened full-texts of selected studies against the inclusion and exclusion criteria. During all stages of study selection, any uncertainties or discrepancies were discussed until consensus was achieved. If consensus was not reached, disagreements were resolved by discussing them with senior researchers (FFL, PV, IC and CT).

### 2.3. Data extraction

The following variables were extracted and entered into a standard data extraction form: author, publication year, country treating hospital, study type, number of included patients, GA at time of fetal procedure, type of disease, fetal procedure performed, anesthesia modality (both maternal and fetal), anesthetic drugs used, duration of procedure, maternal and fetal perioperative complications, gestational age at delivery, procedure outcome and effectiveness.

### 2.4. Synthesis

Fetal procedures were addressed as effective whenever they led to the delivery of vital neonates. Exception was the MMC repair, in which the reversal of the hindbrain herniation was considered as additional criteria. Referring to the laser ablation of placental anastomosis (in twin-to-twin transfusion syndrome), effectiveness was assumed when both fetuses survived.

Perioperative complications were divided between intraoperative, maternal postoperative, and fetal postoperative complications. Maternal postoperative complications were gathered into five groups: chorioamnionitis, abruptio placentae, chorioamniotic membrane separation, acute respiratory distress syndromes and “other”. All postoperative fetal complications were independently considered.

Premature rupture of membranes (PROM) and perinatal deaths (within the first 24 h) were considered as separate outcomes. When evaluating the proportion of perinatal deaths on the total number of patients, twin and triplet pregnancies were considered two and three times, respectively. Miscarriages and termination of pregnancies were considered as perinatal deaths. Additional outcomes evaluated were the length of hospitalization (LOH) after the fetal intervention and GA at delivery.

In order to verify whether the anesthesia modality has an influence on the procedure outcome, subgroups analysis was performed evaluating maternal and fetal anesthesia (MFA) vs. maternal anesthesia (MA) only. Similar evaluations were performed for the most represented fetal procedures. Additionally, considering only the procedures requiring invasive fetal manipulation (MMC repair, FETO, shunting, resection of sacrococcygeal teratoma, ligation of amniotic bands, balloon valvuloplasty/septoplasty, and ovarian cyst resection/aspiration), further evaluations on the impact of fetal anesthesia were conducted. Lastly, the most commonly used anesthetic agents for both, general maternal anesthesia and fetal anesthesia, were described.

### 2.5. Statistical analysis

Aggregated continuous baseline variables were calculated as means or medians of extracted variables from the included studies. Categorical and continuous variables were summarized as numbers with percentages. Statistical analysis was performed using Fisher's exact test for categorical data, while continuous data were compared using Mann Whitney U-test. *P* <0.05 was considered statistically significant.

## 3. Results

**Figure 1:**
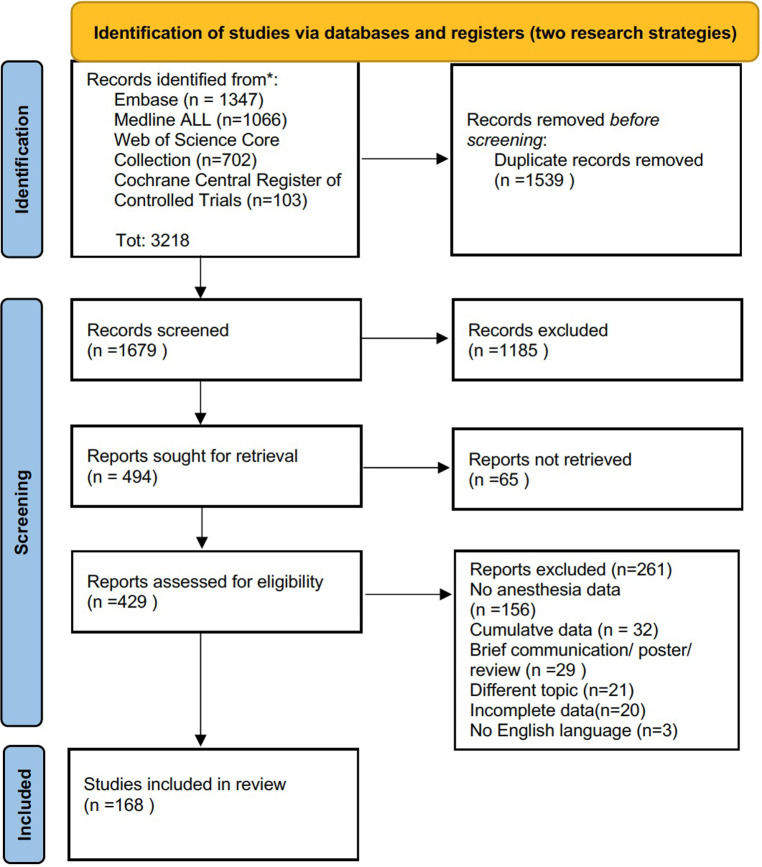
PRISMA flow diagram of literature search (10).

The systematic search strategies yielded 1,679 articles, and 429 of them were further assessed for eligibility. After full-text screening against inclusion criteria, 168 articles, accounting for 6,761 procedures, were selected (Figure 1) (11–178). Fourteen of the included studies, not providing information on the procedure outcome, were only considered for the initial description of anesthesia modality and in the summary of anesthetic drugs used (13, 25, 31, 42, 53, 60, 66, 109, 110, 123, 142, 147, 148, 165). Characteristics of the included studies can be found in Supplementary Material.

### 3.1. Anesthesia modality

A total of 165 studies reported detailed information on the anesthesia modality, accounting for 6,751 patients. In 106 of them (5,087 procedures) only MA was provided, while MFA was delivered in 64 studies (1,664 procedures) (1–13, 14–69, 71–123, 125–178). Fetal anesthesia was more frequently administered in case of general maternal anesthesia (*p* = 0.0001).

#### 3.1.1. Maternal anesthesia

Ninety-seven studies, accounting for 4,070 pregnancies, were included in the baseline characteristics and outcome analysis for MA (11, 15, 21–24, 26–29, 33, 35, 38–40, 46, 48, 50–52, 58, 61–65, 67–69, 71–82, 84, 87, 89–94, 96, 97, 99, 101–103, 105, 107, 111, 113–117, 120, 122, 126, 128–134, 137–139, 149, 151, 152, 154–157, 159–162, 164, 166–169, 171, 173–175, 178). The aggregated mean GA at procedure, based on data from 85 studies (3,801 pregnancies), was 27.03 weeks (11, 15, 22–24, 26–29, 33, 35, 38, 40, 46, 48, 50–52, 58, 61–63, 65, 67–69, 71–82, 87, 89, 90, 92–94, 96, 97, 99, 101–103, 105, 107, 111, 113, 116, 117, 120, 122, 126, 128–134, 137, 138, 149, 151, 154–157, 160, 162, 164, 166–169, 171, 173, 175, 178). The mean LOH resulted to be 4.98 days, based on 24 studies involving 903 women (21, 24, 28, 29, 40, 46, 52, 64, 73, 77, 84, 90, 91, 93, 99, 105, 114, 115, 120, 134, 137, 149, 155, 173). Sixty-four studies, with 3,032 pregnancies, resulted in an aggregated mean GA at delivery of 33.39 weeks (11, 22, 27–29, 33, 39, 40, 46, 50, 51, 58, 61, 64, 65, 69, 73–78, 80, 81, 87, 90–93, 94, 96, 99, 101, 102, 105, 107, 113–117, 120, 122, 130–134, 137–139, 152, 154–157, 159, 160, 164, 166–169, 171, 173, 175, 178). Overall, PROM occurred in 888 pregnancies (21.8%). A total of 1,493 fetuses died during pregnancy or within the first 24 h of life (21.45%). The procedures were reported as effective in 73.99% of cases.

A total of 44 intraoperative complications were reported (1.08%), while fetal postoperative complications were observed in 77 cases (1.89%). Maternal postoperative complications were encountered in 332 women (8.15%), consisting of 115 chorioamniotic membrane separations, 104 abruptio placentae, 54 chorioamnionitis, 17 acute respiratory distress syndromes, and 42 “other”. Complications’ details can be found in Supplementary material.

##### 3.1.1.1. Loco-regional vs. general maternal anesthesia

Ninety-five studies (3,905 pregnancies) were included for the comparative analysis of the anesthesia administration's routes (11, 15, 21–24, 26–29, 33, 35, 38–40, 46, 48, 50–52, 58, 61–65, 67–69, 71–82, 84, 87, 89–94, 96, 97, 99, 101–103, 105, 107, 111, 113–116, 120, 122, 126, 128–134, 137–139, 149, 151, 152, 154–157, 159–162, 164, 166–169, 171, 173–175). No statistically significant differences were found in the baseline characteristics and outcomes between patients receiving loco-regional and general anesthesia (Table 1).

**Table 1:** Baseline characteristics and outcomes for loco-regional anesthesia vs general anesthesia in procedures performed under maternal anesthesia.

	*N* studies (*N* pt)	GA procedure *N* Studies (*N* pt)	Mean GA at procedure (weeks)	LOH *N* Studies (*N* pt)	Mean LOH (days)
Loco-regional	42 (2572)	34 (2312)	24.37	5 (83)	2.2
General	60 (1328)	55 (1169)	28.15	19 (670)	5.65

Pt, patients; GA, Gestational age; LOH, Length of hospitalization.

#### 3.1.2. Maternal and fetal anesthesia

MFA was provided in 1,551 procedures from 59 studies (12, 16–20, 30, 32, 34, 36, 37, 41, 43–45, 47, 49, 50, 52, 54–57, 59, 62, 63, 68, 83, 85, 86, 88, 95, 98, 100, 104, 106, 108, 112, 118, 119, 121, 125, 127, 136, 140, 141, 143–146, 150, 153, 158, 163, 170, 172, 176, 177). Fetal anesthesia administration was specified in 50 studies, consisting of 635 intramuscular injections (45 studies), 292 subcutaneous injections (three studies) and four intravenous injections (two studies) (16–20, 30, 32, 34, 36, 37, 41, 43, 44, 47, 49, 50, 52, 54, 55, 57, 59, 63, 83, 86, 95, 98, 100, 104, 106, 108, 118, 119, 121, 125, 127, 136, 140, 141, 143–146, 150, 153, 158, 163, 170, 176, 177). An aggregate mean GA at procedure of 27.99 weeks was calculated on 49 studies and 1,404 pregnancies (12, 16–19, 30, 32, 34, 36, 37, 41, 43–45, 47, 49, 50, 52, 54–57, 59, 62, 63, 68, 83, 85, 86, 88, 95, 98, 100, 104, 106, 108, 112, 118, 119, 121, 125, 127, 135, 136, 140, 141, 143–146, 150, 153, 158, 163, 170, 172, 176, 177). Mean LOH, based on data from 18 studies and 92 women, was 3.79 days (16, 18, 19, 32, 36, 43–45, 47, 52, 55, 86, 98, 100, 106, 118, 153, 176). A total of 33 studies (1,196 pregnancies) provided information on the GA at delivery, resulting in an aggregate mean of 34.89 weeks (17–19, 37, 41, 45, 49, 54, 59, 108, 118, 121, 125, 127, 135, 136, 140, 141, 143–145, 158, 170, 176, 177). PROM occurred in 401 pregnancies (25.85%) and 197 fetal deaths were reported (11.37%). Overall, effectiveness of the procedures was 75.85%.

Complications during the procedure occurred in 12 cases (0.74%). Postoperative complications, either fetal or maternal, were reported for 27 (1.6%) and 174 (10.8%) procedures, respectively. Maternal complications comprised 121 chorioamniotic membrane separation, 25 chorioamnionitis, twelve abruptio placentae, three acute respiratory distress syndromes, and thirteen “other”. Complications’ details can be found in Supplementary material.

##### 3.1.2.1 Loco-regional vs. general maternal anesthesia in MFA

All studies providing MFA were included in the comparison. The mean LOH resulted to be significantly longer in women who underwent general anesthesia, compared to those who underwent loco-regional anesthesia (*p* = 0.0088) (16, 18, 19, 32, 36, 43–45, 47, 52, 55, 86, 98, 100, 106, 118, 153, 176). No other statistically significant differences were found (Table 2).

**Table 2:** Baseline characteristics and outcomes in maternal loco-regional anesthesia vs general anesthesia in procedures performed under maternal and fetal anesthesia.

	*N* studies (*N* pt)	GA procedure *N* Studies (*N* pt)	Mean GA at procedure (weeks)	LOH *N* Studies (*N* pt)	Mean LOH (days)
Loco-regional	24 (742)	22 (690)	26.93	6 (32)	1.33
General	36 (809)	28 (653)	28.97	12 (60)	5.23

Pt, patients; GA, Gestational age; LOH, Length of hospitalization.

### 3.2. Laser ablation of placental anastomosis

Twenty-nine studies, accounting for 2,964 pregnancies with twin-to-twin transfusion syndrome (TTTS), were included (22, 27, 48, 61, 64, 69, 72, 79, 87, 94, 96, 102, 105, 107, 114, 117, 122, 131–133, 149, 155, 156, 164, 167–169, 174). All but one study performed MA; the one study describing laser ablation of placental anastomosis under MFA was excluded from the sub analysis (135). Patients baseline characteristics and procedure outcomes were compared between the administration of loco-regional and general MA, without identifying any statistical difference (Table 3).

**Table 3:** Baseline characteristics and outcomes in maternal loco-regional anesthesia vs general anesthesia in laser ablation of placental anastomosis procedures.

	*N* studies (*N* pt)	GA procedure *N* Studies (*N* pt)	Mean GA at procedure (weeks)	LOH *N* Studies (*N* pt)	Mean LOH (days)	DOP *N* studies (*N* pt)	Mean DOP (min)	GA at delivery *N* studies (*N* pt)	Mean GA at delivery (weeks)	PROM *N*/*N* FOP (%)	FD *N*/*N* pt (%)	EF *N* Studies (*N* pt)^a^	EF *N*/*N* pt^a^ (%)
Loco-regional	24 (2120)	21 (1865)	21.95	3 (81)	2.33	10 (832)	39.68	16 (687)	31.92	217/1,430 (15.17)	797/2,861 (27.85)	19 (1271)	811/1,271 (63.80)
General	8 (596)	8 (596)	20.97	1 (70)	2.4	3 **++** (177)	70.87	3 (177)	30.47	22/244 (9)	105/488 (21.51)	4 (244)	173/244 (70.9)

Pt, patients (1,429 twin pregnancies, one triplet pregnancy); GA, Gestational age; LOH, Length of hospitalization; DOP, duration of procedure; FD, Fetal death; EF, Effectiveness of procedure.

^a^ Total number of patients for whom effectiveness was specified.

### 3.3. Myelomeningocele repair

MMC repair was described in 35 studies, totaling 1,372 patients. Open (16, 20, 24, 28, 40, 46, 54, 73, 85, 86, 91, 113, 115, 120, 121, 127, 137, 154, 156, 158, 166, 172, 176, 177) and fetoscopic (17–19, 28, 29, 33, 99, 134, 140, 171, 178) repair were individually evaluated. One study was excluded due to cumulative data on both surgical approaches (41). For each operative technique, baseline characteristics and procedure outcomes were evaluated based on the provision of MA or MFA. In both analysis there were no statistically significant differences (Tables 4, 5).

**Table 4:** Baseline characteristics and outcomes in maternal general anesthesia vs maternal and fetal anesthesia in open myelomeningocele repair.

Open MMC repair	*N* studies (*N* pt)	GA procedure *N* studies (*N* pt)	Mean GA at procedure (weeks)	LOH *N* Studies (*N* pt)	Mean LOH (days)	DOP *N* studies (*N* pt)	Mean DOP (min)	GA at delivery *N* studies (*N* pt)	Mean GA at delivery (weeks)	PROM *N*/*N* FOP (%)	FD *N*/*N* pt (%)	EF *N* Studies (*N*pz)	EF *N*/*N* pt (%)
General MA	13 (549)	12 (548)	25.18	9 (484)	7.55	6 (406)	139.04	12 (504)	34.27	122/549 (22.2)	22/549 (4)	13 (549)	487/549 (88.7)
MFA	11 (571)	8 (430)	24.28	3 (16)	7.5	4 (298)	127.43	7 (439)	34.89	134/571 (23.34)	15/571 (2.63)	10 (557)	400/557 (71.81)

MA, Maternal anesthesia; MFA, Maternal-fetal anesthesia; GA,gestational age; LOH, length of hospitalization; DOP, duration of procedure; PROM, premature rupture of membranes; FOP, fetal operative procedures; FD, Fetal death; EF, Effectiveness of procedure; Pt = patients.

**Table 5:** Baseline characteristics and outcomes in maternal general anesthesia vs maternal and fetal anesthesia in fetoscopic myelomeningocele repair.

Fetoscopic MMC repair	*N* studies (*N* pt)	GA procedure *N* studies (*N* pt)	Mean GA at procedure (weeks)	LOH *N* Studies (*N* pt)	Mean LOH (days)	DOP *N* studies (*N* pt)	Mean DOP (min)	GA at delivery *N* studies (*N* pt)	Mean GA at delivery (weeks)	PROM *N*/*N* FOP(%)	FD *N*/*N* pt (%)	EF *N* Studies (*N*pz)	EF *N*/*N* pt (%)
**General MA**	7 (134)	7 (134)	25.27	4 (63)	3.9	4 (23)	258.55	7 (134)	31.62	49/134 (36.56)	5/134 (3.73)	7 (134)	124/134 (92.53)
**MFA**	4 (27)	4 (27)	24.74	2 ++ (23)	5	2 ++ (23)	245	4 (27)	34.68	7/27 (25.9)	0/27	4 (27)	17/27 (62.96)

GA, gestational age; MA,  Maternal anesthesia; MFA, Maternal-fetal anesthesia; LOH, length of hospitalization; DOP, duration of procedure; PROM, premature rupture of membranes; FOP, fetal operative procedures; FD, Fetal death; EF, Effectiveness of procedure; pt, patients.

### 3.4. Fetoscopic endoluminal tracheal occlusion

A total of 508 fetuses with CDH, from twelve studies, underwent FETO (44, 50, 51, 56, 78, 88, 135, 136, 141, 143, 146, 172). In 58 cases only MA was performed (50, 51, 78). Aggregate mean GA at procedure and at delivery were 26.5 and 32.8 weeks, respectively. The procedure resulted effective in 91.4% of cases, with 30 PROM and 7 fetal deaths being described.

MFA was provided in nine studies and 450 procedures (44, 56, 88, 135, 136, 141, 143, 146, 172). Seven studies reported the GA at procedure, which resulted in an aggregate mean of 27.3 weeks (44, 56, 88, 135, 136, 143, 146). Aggregated mean GA at delivery was 34.1 weeks, based on data from six studies (50, 88, 136, 141, 143, 146). PROM and fetal deaths rates were 43.3% and 6.9%, respectively. Overall, effectiveness of the procedure was 79.3%.

### 3.5. Ex-utero intrapartum treatment

Forty-one of the included studies, accounting for 129 patients, described the EXIT procedure (12, 15, 21, 23, 26, 30, 32, 34, 35, 38, 52, 55, 57, 62, 63, 67, 68, 71, 82–84, 89, 92, 95, 97, 98, 100, 103, 104, 106, 111, 119, 124, 126, 128, 129, 151, 153, 160–162). Six studies provided cumulative data on the anesthesia modality and therefore were excluded from the subgroup analysis (21, 38, 62, 68, 124, 129). Studies describing MA or MFA were compared, and no statistically significant differences were found (Table 6).

**Table 6:** Baseline characteristics and outcomes in maternal general anesthesia vs maternal and fetal anesthesia in ex-utero intrapartum treatment procedure.

Anesthesia Modality	*N* studies (*N* pt)	GA procedure *N* Studies (*N* pt)	Mean GA at procedure (weeks)	LOH *N* Studies (*N* pt)	Mean LOH (days)	DOP *N* studies (*N* pt)	Mean DOP (min)	FD *N*/*N* pt (%)	EF *N* Studies (*N* pt)	EF *N*/*N* pt (%)
General MA	21 (26)	19 (22)	35.76	2 (2)	3.5	7 (7)	32.14	3/26 (11.54)	21 (26)	23/26 (88.46)
MFA	16 (86)	12 (82)	34.54	7 (21)	3.97	9 (67)	40.56	5/86 (5.81)	16 (86)	80/86 (93)

GA, gestational age; MA, Maternal anesthesia; MFA, Maternal-fetal anesthesia; LOH, length of hospitalization; DOP, duration of procedure; FD, Fetal death; EF, Effectiveness of procedure; pt, patients.

### 3.6. Shunting

Seven studies, accounting for 39 patients, described a shunting procedure due to pleural effusion (*n* = 2), lower urinary tract occlusion (*n* = 3) and congenital lung malformation (*n* = 2) (11, 39, 75, 145, 152, 170, 175). Maternal anesthesia was performed in eleven patients from five studies (11, 39, 75, 152, 175). The aggregated mean GA at procedure, based on three studies, was 29.6 weeks (11, 75, 175). All studies provided information on GA at delivery, resulting in an aggregate mean of 35.22 weeks. PROM and fetal death rates were 45.45% and 1.8%, respectively; effectiveness of the procedure was 81.8%. Two studies, totaling 28 patients, preferred MFA (145, 170). Aggregated mean GA was 21.65 weeks at procedure, and 31.6 weeks at delivery. Fetal deaths occurred in 53.5% of cases; the procedure effectiveness was 43.75%.

### 3.7. Impact of fetal anesthesia in procedures requiring invasive fetal manipulation

To evaluate the impact of fetal anesthesia, 81 studies, accounting for 2,112 patients, were considered. MA was performed in 827 procedures (11, 24, 28, 29, 33, 39, 40, 46, 50, 51, 58, 65, 73, 75–78, 81, 90, 91, 99, 101, 113, 115, 120, 130, 134, 137–139, 152, 154, 157, 159, 166, 171, 173, 175, 178), while MFA in 1,285 (14, 16–20, 36, 37, 41, 43–45, 47, 49, 50, 54, 56, 59, 70, 85, 86, 88, 108, 112, 118, 121, 125, 127, 135, 136, 140, 141, 143–146, 150, 158, 163, 170, 172, 176, 177).

PROM and fetal deaths showed a higher prevalence in the MFA group (*p* < 0.0001 and *p* = 0.0169, respectively). Subsequently, the procedures performed under MA resulted to be more effective (88.63% vs. 74.57%, *p* < 0.0001). The remaining parameters did not show any statistical differences (Table 7).

**Table 7:** Outcomes comparison in maternal anesthesia vs maternal and fetal anesthesia in procedures requiring invasive fetal manipulations.

Anesthesia Modality	*N* studies (*N* pt)	LOH *N* Studies (*N* pt)	Mean LOH (days)	GA at delivery *N* studies (*N* pt)	Mean GA at delivery (weeks)	PROM *N*/*N* FOP (%)	FD *N*/*N* pt (%)	EF *N* Studies (*N* pt)	EF *N*/*N* pt (%)
MA	39 (827)	15 (596)	5.7	37 (781)	33.92	38/827 (4.59)	47/827 (5.68)	39 (827)	733/827 (88.63)
MFA	43 (1285)	11 (71)	3.68	33 (1016)	35.16	375/1,346 (27.86)	109/1,346 (8)	40 (1292)	918/1,292 (71)

GA, gestational age; MA, Maternal anesthesia; MFA, Maternal-fetal anesthesia; LOH, length of hospitalization; PROM, premature rupture of membranes; FOP, fetal operative procedures; FD, Fetal death; EF, Effectiveness of procedure; pt, patients.

### 3.8. Fetal and maternal anesthetic agents

#### 3.8.1. Anesthetic agents for fetal anesthesia

Sixty studies, accounting for 1,524 procedures, described the anesthetic agents used to provide direct fetal anesthesia (14, 16–20, 30, 32, 34, 36, 37, 41, 43–45, 47, 49, 50, 52, 54–57, 59, 62, 63, 66, 68, 70, 83, 85, 86, 88, 95, 98, 100, 104, 106, 108–110, 118, 121, 125, 127, 135, 140, 141, 143–148, 153, 158, 163, 170, 176, 177). The most common anesthetic agents are summarized in Table 8.

**Table 8:** Most commonly used fetal anesthetic agents.

	*N* Studies	*N* patients	Dosage µg/Kg
Fentanyl	48	1501	0.2–20,000
Atropine	31	820	0.2–200
Vecuronium	26	487	0.2–400
Pancuronium	12	455	0.3–2000

#### 3.8.2. Anesthetic agents for general maternal anesthesia

Fifty-six studies, with a total of 1,127 patients, specified the drugs used to provide general maternal anesthesia (11, 13, 15, 19, 22, 24–26, 30–32, 35, 41, 42, 46, 50, 52, 53, 55, 57, 63, 64, 66, 71, 73, 78, 81, 82, 84, 86, 90, 95, 97, 99, 103, 104, 106, 108–110, 115, 119, 124, 126, 128, 134, 141, 142, 151, 153, 158, 160–162, 173). The extreme variability between the studies did not allow a specific analysis. The preferably used induction agents for general MA included thiopental, associated with neuromuscular blocking drugs (e.g., succinylcholine, rocuronium) or propofol. Following, MA was maintained with either a volatile anesthetic agent (e.g., desflurane, sevoflurane, isoflurane) or a combination of volatile and intravenous (e.g., propofol) anesthetic agents. Seldom, an epidural catheter was inserted for postoperative analgesia.

## 4. Discussion

### 4.1. Summary of main results

The studies included in this systematic review widely varied in terms of study design, population and outcome. Several anesthesia approaches were used, with no standardized protocols nor common strategies based on the fetal procedure.

Overall, no significant differences were found among procedures performed under MA or MFA, reflecting the absence of a close link between reported outcomes and anesthesia modality. Complicating furthermore, several factors influencing the procedures’ outcome (e.g., maternal comorbidities, pregnancy-related health conditions, etc.) were not systematically reported.

Procedures requiring invasive fetal manipulation resulted to be more effective when performed under MA only. Indeed, higher rates of PROM and fetal deaths were found in the MFA group. Based on the retrieved data, the combined use of maternal and fetal anesthetics could translate in fetal over treatment and, consequently, greater risk of fetal death. Additionally, fetal direct anesthesia mostly involved the administration of curare, which exposure-related effects on fetuses have not been fully understood yet. Nevertheless, further studies are necessary to properly evaluate such results and identify an eventual physiopathological explanation.

Lastly, a significant preference for the use of fentanyl, atropine, vecuronium or pancuronium was found in the provision of direct fetal anesthesia. Anyhow, dosages used in different studies were extremely heterogeneous, varying among hundreds of micrograms. The same conclusions can be drawn referring to maternal general anesthetics, thus only few studies reported dosages for the maternal drugs used. Based on the available data, no specific anesthetic modality proved to be superior to the others.

### 4.2. Potential biases in the review process

The quality of the available evidence on the maternal-fetal anesthesia management during fetal surgery is poor. Although included studies provided some anesthesia data, most of them aimed to describe the surgical technique or the effectiveness of the procedure as primary outcome. As a consequence, anesthesia details were missing, as those referring to intraoperative monitoring. Indeed, only few studies included information regarding intraoperative fetal heartbeat variations or fetal movements. Hence, defining the impact of anesthesia on the procedure's performance was not possible.

Additionally, in order to incorporate a large group of patients for this systematic review, studies with wide variance in terms of methodology were included, and no limitation on publication date was defined. As a consequence, the overall quality of the studies might have been negatively influenced.

Complicating furthermore, fetal surgical procedures became more popular over time and several authors who started with a case report description are now sharing their experience on a large cohort of patients. This translates in a potential population bias, with the inclusion of some patients more than once.

### 4.3. Agreements and disagreements with other studies or reviews

The need for adequate MFA during fetal interventions is a highly debated topic. Indeed, the ever-increasing performance of invasive prenatal surgery brought some authors to address concerns on the fetuses’ pain perception. Studies on intraoperative fetal monitoring revealed that neuroinhibiting substances (adenosine, pregnanolone, prostaglandine D2) (179), ensuring a continuous sleep status during pregnancy, are inadequate to ensure fetal anesthesia (7). Subsequently, starting from the second trimester, fetuses seem to be awakened by external stimuli (180).

Additionally, current evidence highlights how an early form of pain could appear in fetuses starting from 15 weeks of GA, mainly depending on the reticular formation of the mesodiencephalon. Later on, the diencephalon seems to occupy a leading role in the fetal pain experience and, only toward the end of pregnancy, the nociceptive pathway is completed by the cortex cerebri development (6).

Nevertheless, exact pain processing pathways during fetal life are yet to be extensively evaluated; and whether an external stimuli is able to trigger a conscious cortical processing is still debated. As a consequence, no standardized recommendations for anesthesia during FOP are available.

Some authors consider fetal direct anesthesia to be justified, as it provides intraoperative fetal immobilization and, at the same time, ensures no pain perception (181). This way, it might avoid the long-term impact of early painful experiences, which proved to alter the course of sensory development (182). Conversely, other authors have concerns on the potentially negative effects of direct early-life exposure to general anesthetics. Neurotoxicity or behavioral and cognitive deficits have been previously demonstrated, yet only a few longitudinal studies on this topic are available (183, 184). Adding up to this, some studies proved that fetal immobilization can be obtained through maternal sedative drugs administration (e.g., diazepam or remifentanil), overcoming the need for combined MFA (185).

Results from this systematic review do not provide an answer to this controversy. Anyhow, based on the retrieved data, anesthesia modality seems to have no impact on the fetal procedure's outcome. The great variety of anesthetic approaches used, even when comparing the same procedure, does not translate into different perioperative or delivery-related complication rates. Nevertheless, when comparing anesthesia modalities for invasive fetal procedures, interventions involving direct fetal anesthesia resulted in a worse outcome (higher PROM and fetal deaths rates). This result highlights the need for future evaluation on early-life anesthetic administration, bearing in mind that the retrieved data are insufficient to entirely prove a causal association between the two events.

Another point to consider is that no study fully analyzed fetal reactions to the surgical procedure, complicating the choice of outcome parameters to use in order to evaluate the impact of different anesthesia modalities. Indeed, the outcome criteria used in this review are not uniquely linked to the anesthetic approach, yet influenced by several fetal and maternal factors.

Ring / Ginosar and Van de Velde et al. provided some general suggestions on the anesthesia modality to be used, based on the invasiveness and fetal direct manipulation of different FOP's categories (182, 186). However, the real need for fetal direct anesthesia has not been properly assessed and, based on this systematic review, no study explicitly takes into account the transplacental passage of anesthetics while providing MFA.

Referring to the anesthetic drugs used, included studies describing fetal direct anesthesia mostly administered fentanyl, atropine, and vecuronium/pancuronium. This is in line with previous reports and necessary to obtain complete fetal immobilization (186).

Conversely, maternal anesthesia did not follow a specific pattern nor required the use of particular drugs. The involved studies widely varied in terms of maternal anesthetic agents, most likely depending on each center policy. Worth mentioning, more recent studies showed a trend toward new maternal anesthetic drug, combinations aiming to ensure adequate uterine relaxation. This is in line with the American consensus statement on anesthesia for maternal-fetal intervention (187). Indeed, high doses of volatile anesthetic agents were traditionally used to maintain uterine relaxation. However, this practice can be associated with significant fetal bradycardia. More recently, intravenous anesthesia with remifentanil allowed to reduce the dosages of volatile anesthetics and minimize fetal cardiac dysfunction.

### 4.4. Implication for practice

One of the primary aims of this systematic review was to evaluate the impact of different anesthetic approaches on FOP's outcome. Due to the heterogeneity of the included studies and the lack of standardized intraoperative fetal monitoring, defining the best anesthesia modality between MA and MFA is difficult. When referring to the procedures' outcome, and based on the retrieved data, fetal direct anesthesia does not seem to be an added value in FOP, not positively influencing neither perioperative complications nor fetal deaths rates.

Nonetheless, recent literature on fetal pain perception opens up to a pathophysiological and ethical discussion, encouraging the use of MFA. Although fetal direct anesthesia might not be technically essential, it might be paramount for the fetal wellbeing and neurological development.

### 4.5. Implication for research

This systematic review highlights the lack of standardized anesthetic approaches to FOP. The ongoing improvement of prenatal surgical care requires a parallel implementation of anesthesia guidelines and protocols. Further studies aiming to evaluate fetal reaction to pain and comparing different anesthesia approaches are needed. Fetal intraoperative parameters, together with hormonal responses to different stimuli and anesthetic approaches, should be systematically investigated. Alongside, as long-term neurocognitive impairment has been proved to be caused by early-life exposure to both, anesthetics agents and painful experiences, prospective studies on neurodevelopment for school-aged children who underwent FOP with different anesthetic approaches, might solve the dilemma between MA and MFA.

## 5. Conclusions

This systematic review shows great variance in the anesthetic management for maternal-fetal interventions. Available evidence is too diverse to define the best modality for drug delivery and the optimal drug to be used for these procedures. Further studies systematically reporting intraoperative fetal monitoring (e.g., heartbeat variations, fetal movements) and fetal hormonal responses to external stimuli are necessary to identify the best anesthetic approach. Moreover, a standardized reporting of such parameters might help evaluate fetal response to pain, and serve as a basis to better understand fetal pain perception. Afterwards, expert consensus would be advisable to improve both maternal and fetal outcomes.

## Data Availability

The original contributions presented in the study are included in the article/**Supplementary Material**, further inquiries can be directed to the corresponding author/s.
