# Supplementary material for: Anesthesia for fetal operative procedures: A systematic review
Source: Front Pain Res (Lausanne). 2022 Sep 12;3:935427. doi: 10.3389/fpain.2022.935427 (PMC9554945; doi:10.3389/fpain.2022.935427)
Supplement: Supplementary file 1 [file Table1.docx]

Supplementary Material

# Supplementary Data

**1.1 Search Strategies** up to December 14 2021

**embase.com 577**

('fetus surgery'/de OR ((amnioscope/de OR fetoscopy/de) AND (surgery/de)) OR (((fetus* OR fetal* OR foetus* OR foetal* OR intrauterin* OR intra-uterin* OR fetoscop* OR amnioscop* OR in-uter* OR ex-uter* OR prenatal* OR pre-natal*) NEAR/3 (surg* OR operat* OR procedure* OR intervention* OR repair* OR therap*))):Ab,ti) AND ('anesthesiological procedure'/de OR anesthesia/exp OR anesthesist/de OR anesthesiologist/de OR 'anesthesia complication'/de OR 'anesthetic agent'/de OR 'local anesthetic agent'/de OR (anesthe* OR anaesthe*):Ab,ti) NOT ([animals]/lim NOT [humans]/lim) NOT [conference abstract]/lim AND [english]/lim

**Medline ALL**

(((Fetoscopes/) AND (General Surgery/)) OR (((fetus* OR fetal* OR foetus* OR foetal* OR intrauterin* OR intra-uterin* OR fetoscop* OR amnioscop* OR in-uter* OR ex-uter* OR prenatal* OR pre-natal*) ADJ3 (surg* OR operat* OR procedure* OR intervention* OR repair* OR therap*))).ab,ti.) AND (exp Anesthesia/ OR Anesthesiologists/ OR Anesthetics/ OR Anesthesia, Local/ OR (anesthe* OR anaesthe*).ab,ti.) NOT (exp animals/ NOT humans/) NOT (conference abstract) AND english.la.

**Web of Science Core Collection***

TS=(((((fetus* OR fetal* OR foetus* OR foetal* OR intrauterin* OR intra-uterin* OR fetoscop* OR amnioscop* OR in-uter* OR ex-uter* OR prenatal* OR pre-natal*) NEAR/2 (surg* OR operat* OR procedure* OR intervention* OR repair* OR therap*)))) AND ((anesthe* OR anaesthe*)) NOT (animals NOT humans)) AND LA=(English) AND DT=(Article OR Review OR Letter OR Early Access)

**Cochrane Central Register of Controlled Trials**

((((fetus* OR fetal* OR foetus* OR foetal* OR intrauterin* OR intra NEXT uterin* OR fetoscop* OR amnioscop* OR in NEXT uter* OR ex NEXT uter* OR prenatal* OR pre NEXT natal*) NEAR/3 (surg* OR operat* OR procedure* OR intervention* OR repair* OR therap*))):Ab,ti) AND ((anesthe* OR anaesthe*):Ab,ti) NOT (animals NOT humans) NOT (conference abstract)

**embase.com 768**

('fetus surgery'/mj OR ((amnioscope/mj OR fetoscopy/mj) AND (surgery/mj)) OR (((fetus* OR fetal* OR foetus* OR foetal* OR intrauterin* OR intra-uterin* OR fetoscop* OR amnioscop* OR in-uter* OR ex-uter* OR prenatal* OR pre-natal*) NEAR/3 (surg* OR operat* OR procedure* OR intervention* OR repair* OR therap*))):Ti) AND ('intrauterine blood transfusion'/mj OR 'twin twin transfusion syndrome'/mj OR 'tracheal occlusion'/mj OR 'fetoscopic endoluminal tracheal occlusion'/mj OR 'congenital diaphragm hernia'/mj OR 'thoracoamniotic shunt'/mj OR 'cystic adenomatoid malformation'/mj OR 'vesicoamniotic shunt'/mj OR 'urinary tract obstruction'/mj/exp OR 'neural tube defect'/mj OR meningomyelocele/mj OR 'sacrococcyx teratoma'/mj OR 'amnion band syndrome'/mj OR 'transluminal valvuloplasty'/mj OR 'aortic stenosis'/mj/exp OR 'pulmonary valve stenosis'/mj OR 'ex utero intrapartum treatment'/mj OR 'ovary cyst'/mj OR 'twin reversed arterial perfusion sequence'/mj OR 'multifetal pregnancy reduction'/mj OR (((fetus* OR fetal* OR foetus* OR foetal* OR intrauterin* OR intra-uterin*) NEAR/3 transfus*) OR ((twin-twin OR twin-to-twin) NEAR/3 (transfus* OR syndrome*)) OR (placent* NEAR/3 anastomo*) OR (Fetoscopic NEAR/3 Endoluminal NEAR/3 Trachea* NEAR/3 Occlus*) OR (congenital* NEAR/3 diaphragm* NEAR/3 hernia*) OR ((thoracoamniotic* OR thoraco-amniotic* OR vesicoamniotic* OR vesico-amniotic*) NEAR/3 shunt*) OR (cyst* NEAR/3 adenomat* NEAR/3 malform*) OR ((urinar* OR uret*) NEAR/3 obstruct*) OR (neural-tube* NEAR/3 defect*) OR meningomyelocel* OR meningo-myelocel* OR ((sacrococcyx*) NEAR/3 teratoma*) OR (Ligat* NEAR/3 amniotic-band*) OR (amnion NEAR/3 band NEAR/3 syndrome) OR ((transluminal* OR trans-luminal* OR balloon*) NEAR/3 (valvuloplast* OR septoplast*)) OR ((aort* OR pulmonar*) NEAR/3 steno*) OR (ex-utero NEAR/3 (intrapart* OR intra-part*) NEAR/3 (procedure* OR treatment*)) OR exit-procedure OR (ovar* NEAR/3 cyst*) OR (twin-revers* NEAR/3 arter* NEAR/3 perfus*) OR (reduction NEAR/3 (monochorionic* OR bicohorionic* OR mono-chorionic* OR bi-cohorionic* OR multifetal* OR multi-fetal*))):ti) NOT ([animals]/lim NOT [humans]/lim) NOT [conference abstract]/lim AND [english]/lim

**Medline ALL**

(((* Fetoscopes/) AND (* Surgery/)) OR (((fetus* OR fetal* OR foetus* OR foetal* OR intrauterin* OR intra-uterin* OR fetoscop* OR amnioscop* OR in-uter* OR ex-uter* OR prenatal* OR pre-natal*) ADJ3 (surg* OR operat* OR procedure* OR intervention* OR repair* OR therap*))).ti.) AND (* Blood Transfusion, Intrauterine/ OR * Fetofetal Transfusion/ OR * Hernias, Diaphragmatic, Congenital/ OR * Cystic Adenomatoid Malformation of Lung, Congenital/ OR * Neural Tube Defects/ OR * Meningomyelocele/ OR exp * Aortic Valve Stenosis/ OR Pulmonary Valve Stenosis/ OR * Ex utero Intrapartum Treatment Procedures/ OR * Ovarian Cysts/ OR * Pregnancy Reduction, Multifetal/ OR (((fetus* OR fetal* OR foetus* OR foetal* OR intrauterin* OR intra-uterin*) ADJ3 transfus*) OR ((twin-twin OR twin-to-twin) ADJ3 (transfus* OR syndrome*)) OR (placent* ADJ3 anastomo*) OR (Fetoscopic ADJ3 Endoluminal ADJ3 Trachea* ADJ3 Occlus*) OR (congenital* ADJ3 diaphragm* ADJ3 hernia*) OR ((thoracoamniotic* OR thoraco-amniotic* OR vesicoamniotic* OR vesico-amniotic*) ADJ3 shunt*) OR (cyst* ADJ3 adenomat* ADJ3 malform*) OR ((urinar* OR uret*) ADJ3 obstruct*) OR (neural-tube* ADJ3 defect*) OR meningomyelocel* OR meningo-myelocel* OR ((sacrococcyx*) ADJ3 teratoma*) OR (Ligat* ADJ3 amniotic-band*) OR (amnion ADJ3 band ADJ3 syndrome) OR ((transluminal* OR trans-luminal* OR balloon*) ADJ3 (valvuloplast* OR septoplast*)) OR ((aort* OR pulmonar*) ADJ3 steno*) OR (ex-utero ADJ3 (intrapart* OR intra-part*) ADJ3 (procedure* OR treatment*)) OR exit-procedure OR (ovar* ADJ3 cyst*) OR (twin-revers* ADJ3 arter* ADJ3 perfus*) OR (reduction ADJ3 (monochorionic* OR bicohorionic* OR mono-chorionic* OR bi-cohorionic* OR multifetal* OR multi-fetal*))).ti.) NOT (exp animals/ NOT humans/) NOT (conference abstract) AND english.la.

**Web of Science Core Collection***

TI=(((fetus* OR fetal* OR foetus* OR foetal* OR intrauterin* OR intra-uterin* OR fetoscop* OR amnioscop* OR in-uter* OR ex-uter* OR prenatal* OR pre-natal*) NEAR/2 (surg* OR operat* OR procedure* OR intervention* OR repair* OR therap*)) AND (((fetus* OR fetal* OR foetus* OR foetal* OR intrauterin* OR intra-uterin*) NEAR/2 transfus*) OR ((twin-twin OR twin-to-twin) NEAR/2 (transfus* OR syndrome*)) OR (placent* NEAR/2 anastomo*) OR (Fetoscopic NEAR/2 Endoluminal NEAR/2 Trachea* NEAR/2 Occlus*) OR (congenital* NEAR/2 diaphragm* NEAR/2 hernia*) OR ((thoracoamniotic* OR thoraco-amniotic* OR vesicoamniotic* OR vesico-amniotic*) NEAR/2 shunt*) OR (cyst* NEAR/2 adenomat* NEAR/2 malform*) OR ((urinar* OR uret*) NEAR/2 obstruct*) OR (neural-tube* NEAR/2 defect*) OR meningomyelocel* OR meningo-myelocel* OR ((sacrococcyx*) NEAR/2 teratoma*) OR (Ligat* NEAR/2 amniotic-band*) OR (amnion NEAR/2 band NEAR/2 syndrome) OR ((transluminal* OR trans-luminal* OR balloon*) NEAR/2 (valvuloplast* OR septoplast*)) OR ((aort* OR pulmonar*) NEAR/2 steno*) OR (ex-utero NEAR/2 (intrapart* OR intra-part*) NEAR/2 (procedure* OR treatment*)) OR exit-procedure OR (ovar* NEAR/2 cyst*) OR (twin-revers* NEAR/2 arter* NEAR/2 perfus*) OR (reduction NEAR/2 (monochorionic* OR bicohorionic* OR mono-chorionic* OR bi-cohorionic* OR multifetal* OR multi-fetal*))) NOT (animals NOT humans)) AND LA=(English) AND DT=(Article OR Review OR Letter OR Early Access)

**Cochrane Central Register of Controlled Trials**

((((fetus* OR fetal* OR foetus* OR foetal* OR intrauterin* OR intra NEXT uterin* OR fetoscop* OR amnioscop* OR in NEXT uter* OR ex NEXT uter* OR prenatal* OR pre NEXT natal*) NEAR/3 (surg* OR operat* OR procedure* OR intervention* OR repair* OR therap*))):ti) AND ((((fetus* OR fetal* OR foetus* OR foetal* OR intrauterin* OR intra NEXT uterin*) NEAR/3 transfus*) OR ((twin NEXT twin OR twin NEXT to NEXT twin) NEAR/3 (transfus* OR syndrome*)) OR (placent* NEAR/3 anastomo*) OR (Fetoscopic NEAR/3 Endoluminal NEAR/3 Trachea* NEAR/3 Occlus*) OR (congenital* NEAR/3 diaphragm* NEAR/3 hernia*) OR ((thoracoamniotic* OR thoraco NEXT amniotic* OR vesicoamniotic* OR vesico NEXT amniotic*) NEAR/3 shunt*) OR (cyst* NEAR/3 adenomat* NEAR/3 malform*) OR ((urinar* OR uret*) NEAR/3 obstruct*) OR (neural NEXT tube* NEAR/3 defect*) OR meningomyelocel* OR meningo NEXT myelocel* OR ((sacrococcyx*) NEAR/3 teratoma*) OR (Ligat* NEAR/3 amniotic NEXT band*) OR (amnion NEAR/3 band NEAR/3 syndrome) OR ((transluminal* OR trans NEXT luminal* OR balloon*) NEAR/3 (valvuloplast* OR septoplast*)) OR ((aort* OR pulmonar*) NEAR/3 steno*) OR (ex NEXT utero NEAR/3 (intrapart* OR intra NEXT part*) NEAR/3 (procedure* OR treatment*)) OR exit NEXT procedure OR (ovar* NEAR/3 cyst*) OR (twin NEXT revers* NEAR/3 arter* NEAR/3 perfus*) OR (reduction NEAR/3 (monochorionic* OR bicohorionic* OR mono NEXT chorionic* OR bi NEXT cohorionic* OR multifetal* OR multi NEXT fetal*))):ti) NOT (animals NOT humans)

# Supplementary Tables

**Table 1** Characteristics of included studies

| Author | Year | Country | Study Design | Patients number | Disease | Procedure | Maternal Anesthesia (yes/no) | Fetal Anesthesia (yes/no) |
| --- | --- | --- | --- | --- | --- | --- | --- | --- |
| Adzick S.N et al(11) | 1993 | USA | Case Series | 9 | CCAM | Thoraco-amniotic shunting, excision | yes | no |
| Agarwal A. et al(12) | 2017 | USA | Case Report | 1 | mediastinal mass with pericardial effusion, compression of trachea and esophagus | EXIT | no | yes |
| Arens C et al(13) | 2016 | Germany | Retrospective Study | 59 | MMC | Fetoscopic repair | yes | No |
| Ashmead G.G eta l(14) | 2004 | USA | Case Report | 1 | ureterocele | vescicocentesis | yes | yes |
| Baker P.A. et al(15) | 2004 | New Zeland | Case Report | 1 | severe micrognathia, ear abnormalities and polyhydramnios | EXIT | yes | no |
| Barini R(16) | 2006 | Brazil | Case Report | 1 | MMC | Open repair | yes | yes |
| Baschat A.A et al(17) | 2018 | USA | Case Report | 3 | MMC | Fetoscopic repair | yes | yes |
| Belfort M.A(18) | 2015 | USA | Case Report | 1 | MMC | Fetoscopic repair | yes | yes |
| Belfort M.A(19) | 2017 | USA | Retrospective Cohort study | 22 | MMC | Fetoscopic repair | yes | yes |
| Bennett KA et al.(20) | 2014 | USA | Cohort study | 43 | MMC | open repair | yes | yes |
| Benonis J.G(21) | 2007 | USA | Case Report | 1 | potential airway obstruction (suspected pierre-robin, no test performed) in a vanishing twin pregnancy | EXIT | yes | no |
| Bergh. E.P et al(22) | 2019 | USA | Prospective cohort study | 283 | TTTS | Fetoscopic laser photocoagulation | yes | no |
| Berrington JE et al.(23) | 2010 | UK | Case report | 1 | CDH underwent FETO | EXIT | yes | no |
| Botelho RD et al.(24) | 2017 | Brazil | Case series | 45 | MMC | open repair | yes | no |
| Bouwman C.M.(25) | 2021 | Netherlands | Retrospective study | 4 | Neck mass | EXIT | yes | no |
| Braden A(26) | 2015 | USA | Case Report | 1 | severe fetal micrognathia and retrognathia (+ limb anomalies and congenital heart defects) | EXIT | yes | no |
| Brock CO et al.(27) | 2021 | USA | Case series (prospective) | 475 | TTTS | Fetoscopic laser photocoagulation | yes | no |
| Bruner J.P(28) | 2000 | USA | Case Series | 8 | MMC | Open repair + fetoscopic repair | yes | no |
| Bruner J.P(29) | 1999 | USA | Case series | 4 | MMC | Endoscopic repair | yes | no |
| Bui TH et al.(30) | 2000 | Sweden | Case report | 1 | laryngeal atresia | EXIT | yes | yes |
| Bussey JG(31) | 2004 | USA | Case Series | 11 | TTS | Laser coagulation | yes | No |
| Butwick A et al.(32) | 2009 | USA | Case Report | 1 | cervical teratoma | EXIT | yes | yes |
| Carrabba G et al.(33) | 2019 | Italy | Case Series | 5 | MMC | fetoscopic repair | yes | no |
| Castillo F et al.(34) | 2007 | Spain | Case Report | 2 | cervical teratoma | EXIT | yes | yes |
| Chang CL et al.(35) | 2008 | USA | Case Report | 1 | severe micrognathia | EXIT | yes | no |
| Chmait RH(36) | 2019 | USA | Case report | 1 | cervical mass | Fetal endoscopic tracheal intubation | yes | yes |
| Ciccolo ML et al.(37) | 2012 | USA | Case Report | 1 | mitral regurgitation and aortic stenosis | fetal aortic balloon valvuloplasty | yes | yes |
| Clark K.D(38) | 2004 | USA | Case Report | 1 |  |  | yes | no |
| Clark S.L(39) | 1987 | USA | Case Report | 1 | CCAM + cervical mass | Thoraco-amniotic shunting + EXIT | yes | no |
| Corral E. et al(40) | 2020 | Chile | Case Series | 16 | MMC | Open repair | yes | no |
| Corroenne R. et al(41) | 2020 | USA | Retrospective cohort study | 91 | MMC | 52 fetoscopic, 39 Open repair | yes | yes |
| Cruz-Martinez R et al(42) | 2021 | Mexico | Prospective Cohort study | 44 | MMC | Open repair | yes | no |
| Cruz-Martinez R et al.(43) | 2015 | Mexico | Case report | 1 | cervical mass | Fetal endoscopic tracheal intubation | yes | yes |
| Cruz-Martinez R. et al (44) | 2020 | Mexico | Prospective cohort study | 25 | CDH | FETO | yes | yes |
| Cruz-Martinez R. et al(45) | 2021 | Mexico | Prospective cohort study | 3 | LUTO | fetoscopic urethral meatotomy | yes | yes |
| Cruz-Martinez R. et al(46) | 2021 | Mexico | Prospective cohort study | 60 | spina bifida | open repair /fetoscopic repair | yes | no |
| Cruz-Martinez R. et al(47) | 2015 | Mexico | Case report | 1 | CCAM | fetal bronchoscopy and bronchial membrane laser perforation | yes | yes |
| De Lia JE et al.(48) | 1999 | USA | Case Series | 67 | TTTS | fetoscopic laser occlusion of the chorioangiphagous vessels | yes | no |
| Debska M. et al(49) | 2020 | Poland | Case series | 10 | LUTO | urethroplasty | yes | yes |
| Deprest J. et al(50) | 2004 | UK | Prospective study | 21 | CDH | FETO | yes | 11 yes, 10 no |
| Deprest J.A et al.(51) | 2021 | Belgium | RCT | 40 | CDH | FETO | yes | no |
| Dinges E.(52) | 2018 | USA | Case Report | 2 | severe micrognathia + narrow oropharynx + neck mass | EXIT | yes | 1 yes, 1 no |
| Duron D.V et al(53) | 2013 | USA | Cohort Study | 85 | TTS | Laser ablation | yes | no |
| Elbabaa SK et al.(54) | 2017 | USA | Case Series | 60 | MMC | Open repair | yes | yes |
| Elliot R (55) | 2013 | USA | Case Report | 1 | CHAOS in one twin | EXIT | yes | yes |
| Engels A.C et al(56) | 2014 | Belgium | Cohort study | 141 | CDH | FETO | yes | yes |
| Eschertzhuber S.(57) | 2005 | Austria | Case Report | 1 | cervical mass | EXIT | yes | yes |
| Fan D et al.(58) | 2017 | China | Case Report | 1 | CCAM | in utero resection | yes | no |
| Ferschl M.B(59) | 2016 | USA | Case Report | 1 | severe aortic stenosis | percutaneous fetal aortic balloon valvuloplasty | yes | yes |
| Ferschl MB et al (60) | 2020 | USA | Retrospective study | 136 | Severe aortic stenosis | Fetal aortic valvuloplasty | yes | yes |
| Fichera A. et al.(61) | 2021 | Italy | Case Series | 133 | TTTS | Fetoscopic laser ablation | yes | no |
| Fink R.J (62) | 2011 | USA | Case Report | 3 | fetal goitre, fetal arthrogryposis | EXIT | yes | 2 no, 1 yes |
| Gaiser R.R (63) | 1997 | USA | Case Report | 3 | neck mass, CDH underwent FETO | EXIT | yes | 2 no, 1 yes |
| Galinkin J.L (64) | 2000 | USA | Case Report | 3 | TTTS, acephalic/acardiac fetus in triplet gestation, TRAP | Laser photocoagulation, fetoscopic umbilical cord coagulation of the acephalic/acardiac fetus | yes | no |
| Garcia AM et al.(65) | 1998 | USA | Case Report | 1 | pelvic mass | cyst-amniotic drainage | yes | no |
| Garcia I. et al(66) | 2021 | Spain | Cohort study | 26 | MMC | Fetoscopic repair | yes | yes |
| Garcia PJ et al.(67) | 2011 | USA | Case Report | 1 | cervical mass | EXIT | yes | no |
| George RB et al.(68) | 2007 | USA | Case Series | 3 | multiple skeletal abnormalities | EXIT | yes | 2 no, 1 yes |
| Gil Guevara E. et al(69) | 2017 | USA | Retrospective Study | 86 | TTTS | fetoscopic laser coagulation | yes | no |
| Giorlandino C. et al(70) | 1990 | Italy | Case Report | 1 | ovarian cyst | fine needle cyst aspiration | yes | yes |
| Gonzales S.K et al(71) | 2018 | USA | Case Report | 1 | CHAOS | EXIT | yes | no |
| Greimel P(72) | 2019 | Austria | Retrospective cohort study | 100 | TTTS | Fetoscopic laser ablation or cord occlusion | yes | no |
| Guillbaud L. (73) | 2021 | France | Cohort study | 17 | MMC | Open repair | yes | no |
| Gul A. et Al.(74) | 2008 | Turkey | Case Series | 5 | TRAP | alcohol ablation, bipolar cord coagulation | yes | no |
| Hannah DM et al.(75) | 2020 | USA | Case report | 1 | chylothorax and bilateral pleural effusions | thoracoamniotic shunt | yes | no |
| Hara T et al.(76) | 2021 | Japan | Case series | 4 | ovarian cyst | intrauterine aspiration | yes | no |
| Harrison M.R (77) | 1990 | USA | Case Report | 1 | CCAM | in utero resection | yes | no |
| Harrison MR et al.(78) | 2003 | USA | RCT | 11 | CDH | FETO | yes | no |
| Hecher K. Et al(79) | 1999 | Germany | Retrospective | 73 | TTTS | laser photocoagulation | yes | no |
| Hecher K. et al(80) | 2006 | France | Prospective multicenter study | 60 | TRAP | percutaneous fetoscopic laser coagulation | yes | no |
| Hedrick H.L(81) | 2004 | USA | Retrospective | 4 | Sacrococcygeal theratoma | debulking | yes | no |
| Helfer D.C et al (82) | 2012 | Brazil | Case Reports | 2 | cervical mass , oral tumor | EXIT | yes | no |
| Hirose S. et al (83) | 2004 | USA | Retrospective Study | 52 | Reversal tracheal occlusion for CDH, neck mass, CHAOS | EXIT | yes | yes |
| Hofer I.S et al (84) | 2013 | USA | Case Report | 1 | cervical mass | EXIT | yes | no |
| Horzelska EI et al.(85) | 2020 | Poland | Cohort study | 74 | MMC | Open repair | yes | yes |
| Howley L. et al (86) | 2015 | USA | Retrospective | 14 | MMC | Open repair | yes | yes |
| Ishii K. et al (87) | 2006 | Japan | Case Report | 1 | FFTS in triplet pregnancy | Selective laser photocoagulation of placental communicating vessels | yes | no |
| Jani C. et al (88) | 2009 | Spain | Prospective multicenter study | 210 | CDH | FETO | yes | yes |
| Jayagobi PA et al.(89) | 2015 | Singapur | Case Report | 1 | cervical mass | EXIT | yes | no |
| Johnson M.D et al (90) | 1989 | USA | Case Report | 1 | posterior urethral valves | open bladder marsupialization | yes | no |
| Johnson M.P.(91) | 2003 | USA | Retrospective study | 50 | MMC | Open repair | yes | no |
| Johnson N. et al(92) | 2009 | Canada | Case Report | 1 | Cervical mass | EXIT | yes | no |
| Johnston R et al.(93) | 2014 | USA | Case report | 1 | Type II vasa previa | fetoscopic laser photocoagulation | yes | no |
| Kanazawa S. et al. (94) | 2021 | Japan | Cohort study | 395 | TTTS | Fetoscopic laser surgery | yes | no |
| Kaneko M. et al (95) | 2010 | Japan | Case Report | 1 | Cervical mass | EXIT | yes | yes |
| Kohl T et al. (96) | 2007 | Germany | Case series | 5 | TTTS | Fetoscopic laser ablative surgery | yes | no |
| Kornacki J. et al (97) | 2019 | Poland | Case report | 4 | goitous hypothiroidism, anterior neck mass, neck giant teratoma, CHAOS | EXIT | yes | no |
|  |  |  |  |  |  |  |  |  |
| Laje P et al (98) | 2012 | USA | Case series | 4 | oropharingeal tumors | EXIT | yes | yes |
| Lapa Pedreira D.A et al(99) | 2018 | USA | Cohort study | 45 | spina bifida | fetoscopic repair | yes | no |
| Lazar D.A et al(100) | 2011 | USA | Retrospective study | 12 | giant neck mass | EXIT | yes | yes |
| Lee FL et al.(101) | 2012 | USA | Case report | 1 | CPAM | percutaneous sclerotomy | yes | no |
| Li WF et al. (102) | 2019 | China | Case series | 159 | TTTS | fetoscopic laser therapy | yes | no |
| Liechty K.W et al (103) | 1999 | USA | Case Report | 1 | Cervical mass in one twin | EXIT | yes | no |
| Liechty K.W et al (104) | 1997 | USA | Case series | 5 | cervical mass (3 teratomas, 2 lymphangiomas) | EXIT | yes | yes |
| Lombardo ML et al.(105) | 2011 | USA | Case series | 70 | TTTS | Endoscopic laser ablation | yes | no |
| Luo D et al.(106) | 2015 | China | Case report | 1 | gastroschisis | EXIT for pre-delivery gastroschisis correction | yes | yes |
| Maggio L et al.(107) | 2015 | USA | Cohort study (retrospective) | 92 | TTTS | Fetoscopic laser ablative surgery | yes | no |
| Mallmann MR et al.(108) | 2020 | Germany | Case series | 14 | Aortic stenosis with mitral regurgitation | Atrioseptostomy and aortic valvuloplasty | yes | yes |
| Manrique S. et al (109) | 2019 | Spain | Cohort study | 29 | MMC | Open/Fetoscopic repair | yes | Yes |
| Marsh BJ et al. (110) | 2020 | USA | Case series | 22 | MMC | Open repair | yes | yes |
| Mayer S(111) | 2010 | Germany | Case report | 1 | oral tumor | EXIT | yes | no |
| Mawad W. et al(112) | 2018 | Canada | Case report | 1 | Simple transposition of the great arteries with an intact atrial septum | percutaneous balloon atrial septoplasty | yes | yes |
| Mazzola CA et al. (113) | 2002 | USA | Case report | 3 | MMC | Open repair | yes | no |
| Meriki N et al (114) | 2010 | Australia | Retrospective | 79 | TTTS | selective laser photocoagulation | yes | no |
| Meuli M et al. (115) | 2013 | Switzerland | Case report | 1 | MMC | Open repair | yes | no |
| Middeldorp J.M et al (116) | 2007 | The Netherlands | Prospective Cohort Study | 100 | TTTS | Fetoscopic laser surgery | yes | no |
| Middeldorp J.M et al (117) | 2007 | The Netherlands | Cohort study | 21 | TTTS | Amnio drainage, fetoscopic laser coagulation | yes | no |
| Migliorelli F et al.(118) | 2015 | Spain | Case report | 1 | Lower urinary tract obstruction | Fetoscopic distal urethra coagulation | yes | yes |
| Miwa I. et al(119) | 2012 | Japan | Case Report | 1 | CHAOS | EXIT | yes | yes |
| Moldenhauer JS et al.(120) | 2020 | USA | Cohort study | 264 | MMC | Open repair | yes | no |
| Moron AF et al.(121) | 2018 | Brazil | Cohort study (retrospective) | 236 | Myelomeningocele | Open repair | yes | yes |
| Murata S. et al(122) | 2021 | Japan | Case Report | 1 | TTTS | fetoscopic laser photocoagulation | yes | no |
| Mustafa HJ et al (123) | 2021 | USA | Multicenter study | 411 | TTS | Laser ablation | yes | no |
| Mychaliska G.B et al(124) | 1997 | USA | Case Series | 8 | CDH + hygroma | EXIT | yes | occasionally |
| Noia G et al(125) | 2011 | Italy | Case Series | 13 | Ovarian cyst | cyst drainage | yes | yes |
| Ogamo M. et al (126) | 2005 | Japan | Case Report | 1 | Cervical mass + polyhydramnios | EXIT | yes | no |
| Olejek A et al.(127) | 2020 | Poland | Cohort study | 49 | MMC | Open repair | yes | yes |
| Oliveira E. et al(128) | 2013 | Portugal | Case Report | 1 | cervical malformation | EXIT | yes | no |
| Ossowski K et al.(129) | 2005 | USA | Case report | 1 | conjoined twins | EXIT | yes | no |
| Pang C et al.(130) | 2021 | China | Case series | 5 | pulmonary atresia with intact ventricular septum, critical pulmonary stenosis | fetal pulmonary valvuloplasty | yes | no |
| Papanna R. et al(131) | 2009 | USA | Retrospective cohort study | 48 | TTTS | laparoscopic assisted fetoscopic coagulation, fetoscopic coagulation | yes | no |
| Papanna R. et al(132) | 2013 | USA | Retrospective study | 134 | TTTS | Fetoscopic laser coagulation | yes | no |
| Paramasivam G. et al(133) | 2010 | UK | Retrospective Study | 35 | TTTS | radiofrequency ablation | yes | no |
| Pedreira D.A.L et al(134) | 2016 | Brazil | CT | 10 | MMC | Endoscopic repair | yes | no |
| Peiro JL et al(135) | 2009 | Spain | Case series | 200 | TTTS, lethal anomaly in one twin fetus, CDH, amniotic band | laser coagulation, selective reduction, FETO, amniotic band lysis | yes | yes |
| Persico N et al.(136) | 2017 | Italy | Case series | 21 | CDH | FETO | yes | yes |
| Pruthi V. et al (137) | 2021 | Canada | Case series | 27 | Spina bifida | Open repair | yes | no |
| Quintero R. A et al (138) | 1997 | USA | Case Report | 2 | Amniotic bands | fetoscopic lysis | yes | no |
| Richter J et al.(139) | 2012 | Norway | Case report | 1 | amniotic band | fetoscopic band release | yes | no |
| Riddle S. et al(140) | 2020 | USA | Case Report | 1 | MMC | Fetoscopic repair | yes | yes |
| Rosen M et al(141) | 2003 | USA | Case Report | 1 | CDH | FETO | yes | yes |
| Rossi C et al(142) | 2008 | USA | Retrospective Study | 266 | TTS | Fetoscopic repair | Yes | no |
| Ruano R et al(143) | 2018 | USA | Case Report | 2 | CDH | FETO | yes | yes |
| Ruano R et al. (144) | 2021 | USA | Case Report | 1 | bronchopulmonary sequestration | laser ablation | yes | yes |
| Ruano R. et al(145) | 2015 | Brazil | Cohort Retrospective study | 50 | LUTO | Fetal cystoscopy, vesico-amniotic shunt | yes | yes |
| Ruano R. et al(146) | 2013 | Brazil | Multicenter study | 17 | CDH | FETO | yes | yes |
| Ruano R. et al(147) | 2011 | Brazil | Randomized control trial | 19 | CDH | FETO | yes | Yes |
| Ruano R. et al (148) | 2011 | Brazil | Case-control study | 17 | CDH | FETO | yes | yes |
| Rustico MA et al.(149) | 2012 | Italy | Case series (prospective) | 150 | TTTS | Fetoscopic laser surgery | yes | no |
| Said S.M et al(150) | 2018 | USA | Case Report | 1 | hypoplastic left heart syndrome | US fetal atrial septostomy | yes | yes |
| Sangaletti M(151) | 2021 | Italy | Case Report | 1 | Cervical mass | EXIT | yes | no |
| Schmidt S et al.(152) | 2003 | Germany | Case Report | 1 | Urinary obstruction | fetal cystoscopy and transurethral stent placement | yes | no |
| Schwartz D.A et al(153) | 2001 | USA | Case Report | 1 | intra-oral cyst | EXIT | yes | yes |
| Segura L.G et al (154) | 2020 | USA | Case report | 1 | MMC | open MMC repair | yes | no |
| Sela H.Y et al(155) | 2014 | USA | Case Report | 1 | TTTS | selective laser photocoagulation | yes | no |
| Sepulveda W et al (156) | 2007 | Chile | Retrospective Study | 33 | TTTS | fetoscopic laser coagulation | yes | no |
| Sepulveda W et al. (157) | 2020 | Chile | Cohort study | 58 | MMC | open repair | yes | no |
| Snegovskikh D et al.(158) | 2021 | USA | Case Report | 1 | MMC | Open repair | yes | yes |
| Soothill P.W et al (159) | 2003 | UK | Case report | 1 | LUTO | ultrasound-guided laser treatment | yes | no |
| Stevens G.H et al (160) | 2002 | Netherlands | Case Report | 1 | Neck mass | EXIT | yes | no |
| Subramanian R(161) | 2018 | India | Case report | 1 | cervical mass | EXIT | yes | no |
| Suenaga M et al (162) | 2014 | Japan | Case Report | 1 | micrognathia | EXIT | yes | no |
| Suh E et al. (163) | 2006 | USA | Case report | 1 | aortic stenosis | aortic valvotomy | yes | yes |
| Takano M. et al(164) | 2021 | Japan | Retrospective Cohort study | 26 | TTTS | Fetoscopic laser coagulation | yes | no |
| Tonni G et al(165) | 2019 | Italy | Case Report | 1 | TRAP | Laser photocoagulation | yes | No |
| Tulipan N et al(166) | 1998 | USA | Case Report | 3 | MMC | Open repair | yes | no |
| Valsky DV et al. (167) | 2011 | Spain | Case-control | 352 | TTTS | Fetoscopic laser coagulation | yes | no |
| Ville Y et al.(168) | 1995 | UK | Case series | 45 | TTTS | endoscopic laser coagulation | yes | no |
| Ville Y et al.(169) | 1994 | UK | Case Report | 2 | TRAP | umbilical cord coagulation | yes | no |
| Vinit N et al. (170) | 2019 | France | Retrospective case series | 33 | LUTO | Fetal cystoscopy, vesico-amniotic shunt | yes | yes |
| Volochovic J et al(171) | 2021 | Lithuania | Case Report | 1 | MMC | Fetoscopic repair | yes | no |
| Wilson RD et al.(172) | 2003 | USA | Retrospective case series | 53 | MMC, CCAM, CDH, Sacrococcygeal theratoma | Open repair, resection, FETO, resection | yes | yes |
| Wohlmuth C. et al(173) | 2014 | Austria | Case series | 47 | aortic stenosis, pulmonary atresia, hypoplastic left heart syndrome | aortic valve dilation, pulmonary valve dilation, balloon atrioseptostomy | yes | no |
| Yamamoto M. et al(174) | 2005 | France | Retrospective | 175 | TTTS | Fetoscopic laser coagulation | yes | no |
| Yonemoto H. et al(175) | 2005 | Japan | Prospective | 5 | Fetal pleural Effusion | Thoraco-amniotic shunt | yes | no |
| Zambelli H et al.(176) | 2007 | Brazil | Case Report | 1 | MMC | Open repair | yes | yes |
| Zamlynski M et al.(177) | 2019 | Poland | Cohort study | 49 | MMC | Open repair | yes | yes |
| Ziemann M et al.(178) | 2018 | Germany | Case series | 65 | spina bifida | fetoscopic repair | yes | no |

**Table 2** Complications in procedures performed under maternal anesthesia. TAPS= Twin Anemia Polycythemia sequence; TTS= Twin-to-twin transfusion syndrome; *=Amniotic fluid leakage (n=3), CID with hysterectomy (n=1), Mirror syndrome (n=4), Wound complication (n=4), Paralytic ileus (n=4), Urinary retention and transient Acute Kidney Insufficiency (n=1), Post dural puncture headache (n=1), Bowel herniation (n=1), Oligohydramnios (n=3), Preeclampsia after procedure (n=5), Post-operative bleeding (n=2), Hypertension (n=1 ), Disruption of membrane (n=1), Placental hematoma (n=2).

|  | Type of Complications | N. Patients |
| --- | --- | --- |
| Post-Operative Maternal Complications | Membrane separation | 115 |
|  | Abruptio Placentae | 104 |
|  | Chorioamnionitis | 54 |
|  | Respiratory problems | 7 |
|  | Other | 42 * |
| Intraoperative Complications | Intraoperative Bleeding | 24 |
|  | Cardiac Arrest | 8 |
|  | Iatrogenic Septostomy | 7 |
|  | Trocar dislodgement | 2 |
|  | Conversion to Open Surgery | 1 |
|  | Failure Procedure | 1 |
|  | Fetal Pericardial Effusion | 1 |
| Post-Operative Complications | Recurrence | 37 |
|  | TAPS | 16 |
|  | Septostomy | 12 |
|  | Fetal Anemia | 3 |
|  | Pseudo amniotic bands | 2 |
|  | TTS reversal | 2 |
|  | Cerebral lesion | 1 |
|  | Hydrops | 1 |
|  | Bradycardia | 1 |
|  | Growth Arrest | 1 |
|  | Hydrocephalus | 1 |

**Table 3** Complications in procedures performed under maternal and fetal anesthesia.*= Uterine Rupture (n=5), Preeclampsia (n=4), Vaginal bleeding (n=1), Maternal infection (n=1), Anemia (n=1), Endometritis (n=1).

|  | Type of Complications | N. Patients |
| --- | --- | --- |
| Post-Operative Maternal Complications | Membrane separation | 121 |
|  | Abruptio Placentae | 12 |
|  | Chorioamnionitis | 25 |
|  | Respiratory problems | 3 |
|  | Other | 13 * |
| Intraoperative Complications | Failure Procedure | 4 |
|  | Intraoperative Bleeding | 6 |
|  | Fetal Pleural/Pericardial Effusion | 2 |
| Post-Operative Complications | Recurrence | 14 |
|  | Failure Post procedure | 7 |
|  | Urological Fistula | 3 |
|  | Cardiomegaly | 1 |
|  | Tachycardia | 1 |
|  | Tumor bleeding | 1 |

## Overview of the databases search results

| **Database searched** | **via** | **Years of coverage** | **Records** | **Records after duplicates removed** |
| --- | --- | --- | --- | --- |
| Embase | Embase.com | 1971 - Present | 579 | 219 |
| Medline ALL | Ovid | 1946 - Present | 417 | 413 |
| Web of Science Core Collection | Web of Knowledge | 1975 - Present | 427 | 136 |
| Cochrane Central Register of Controlled Trials | Wiley | 1992 - Present | 90 | 15 |
| **Total** | | | **1513** | **783** |

| **Database searched** | **via** | **Years of coverage** | **Records** | **Records after duplicates removed** |
| --- | --- | --- | --- | --- |
| Embase | Embase.com | 1971 - Present | 768 | 232 |
| Medline ALL | Ovid | 1946 - Present | 649 | 641 |
| Web of Science Core Collection | Web of Knowledge | 1975 - Present | 275 | 19 |
| Cochrane Central Register of Controlled Trials | Wiley | 1992 - Present | 13 | 4 |
| **Total** | | | **1705** | **896** |
